# Fifteen-year mortality trends in Poland analysed with the use of standard expected years of life lost, 2000–2014

**DOI:** 10.1038/s41598-017-09441-5

**Published:** 2017-08-18

**Authors:** Małgorzata Pikala, Irena Maniecka-Bryła

**Affiliations:** Department of Epidemiology and Biostatistics, the Chair of Social and Preventive Medicine of the Medical University of Lodz, Żeligowskiego 7/9, Lodz, Poland

## Abstract

The aim of the study is an evaluation of years of life lost by inhabitants of Poland according to the most important causes of mortality and identification of trends in the period 2000–2014. The study material included a database which contained information gathered from 5,601,568 death certificates of inhabitants of Poland. In order to calculate years of life lost, the SEYLL_p_ index (*Standard Expected Years of Life Lost per living person)* was applied. We also calculated AAPC (*Average Annual Percentage Change*). The SEYLL_p_ index (per 10,000 population) in Poland decreased from 2503.4 in 2000 to 2193.2 in 2014 among males (AAPC = −0.8%, p < 0.05) and from 1430.2 in 2000 to 1269.4 in 2014 among females (AAPC = −0.6%, p < 0.05). In 2014, the top 5 causes of years of life lost were: cardiovascular diseases (721.4 per 10,000 males and 475.6 per 10,000 females), malignant tumours (575.5 and 418.3), unintentional injuries (202.2 and 46.8), intentional injuries (114.6 and 16.3) and digestive diseases (120.2 and 58.3). Due to negative trends, there is a need to implement preventative measures, aimed at reducing mortality caused by respiratory infections in both males and females, malignant tumours in females and diabetes mellitus and intentional injuries in males.

## Introduction

Poland is a country situated in Central Europe. It has over 38 million inhabitants. In 2016, it had the 35th rank worldwide and 6th in the European Union in terms of the population number^1^. The administrative area of Poland amounted to approximately 312 thousand km², which meant the 70th place worldwide and 9th in Europe. Poland was the first member state of the communist block to initiate constitutional, economic and social transformations in 1989, aimed at fostering democracy and a free market^2^.

The Polish health care system is based on an insurance model. According to the Constitution of the Republic of Poland, each citizen is entitled to health care funded from public resources^3^. Citizens are required to pay an obligatory insurance fee amounting to 9% of the personal income, which is transferred to the health insurance institution (the National Health Fund). Certain highly specialist services are funded directly from the Ministry of Health budget.

The economic transformation, which occurred in Poland after 1989, substantially contributed to lifestyle and health behaviours of the Polish society^4, 5^. A health improvement caused by a development of new medical technologies and modern diagnostic methods contributed to many health rates such as a decrease in the death ratio, which in turn, led to an increase in the average life expectancy. In 2000 the average life expectancy was 69.6 years for males and 78.0 years for females. In 2000–2014 the life span for males increased by 4.1 years and for females by 3.7 years, which meant that the average life expectancy in 2014 was 73.7 years for males and 81.7 years for females^6^. Despite these positive trends, the health situation of the Polish population, measured with life expectancy, is still worse than in most European countries. In 2014, among 44 European countries Poland took the 28^th^ position regarding the male life expectancy and the 25^th^ position in terms of the female life expectancy. According to Eurostat, the average life expectancy for Polish male inhabitants was 7.6 years shorter than in Iceland and the average life expectancy for Polish female inhabitants was 4.5 years shorter than for Spanish female inhabitants, whose life expectancy was the longest^7^.

An immediate result of premature mortality is the number of years lost by the population of Poland. Measures used for calculating mortality in units of lost time are more and more often applied in international studies. Many authors believe they are more reliable in showing an economic and social impact of loss connected with premature mortality better than other, commonly used measures^8, 9^. An analysis of particular diseases which contribute to mortality with an application of standardized mortality rates in highly developed countries, reveals that cardiovascular diseases are definitely the most important mortality factor. However, since the above diseases mostly contribute to death in elderly people, social and economic implications are less serious than in the case of factors which contribute to mortality of younger people. An application of measures based on the number of years of life lost allows to detect these differences.

We focus on the application of measures based on years of life lost to assess the epidemiological situation in Poland. We started our research with the Lodz region–one of 16 Polish regions, which is characterised by the highest mortality rates and the shortest life expectancy. We have published several papers on the most common causes of death in the Lodz region, i.e. cardiovascular diseases^10^, malignant tumours^11^, external causes of death^12^, mortality inequalities between urban and rural inhabitants^13^ and mortality trends due to all causes^14^. Extending our scope of research to entire Poland, we dealt with the analysis of selected death causes with relatively low SEYLL values in Poland, but with considerable epidemiological importance on the global scale, i.e. infectious diseases^15^, tuberculosis^16^ and chronic liver disaeases^17^. We have also published the results of our study on years of life lost of the inhabitants of Poland due to the most important death causes in 2011^18^.

This article includes the results of our most recent research on 15-year trends in mortality of the inhabitants of Poland due to 3 broad cause groups: communicable diseases and maternal, perinatal and nutritional disorders (group I), chronic non-communicable diseases (group II) and all injuries (group III) as well as 12 main groups of mortality: cardiovascular diseases, malignant tumours, unintentional injuries, intentional injuries, digestive diseases, mental and neurological conditions, respiratory infections, respiratory diseases, perinatal and infant causes, infections and parasitic diseases, diabetes mellitus and genito-urinary diseases.

## Results

The number of standard expected years of life lost (SEYLL) in Poland in 2000 was 4,297,735 for males and 2,668,117 for females (Table 1). When we compare it to the number of inhabitants (SEYLL_p_), it will amount to 2,503.4 per 10,000 males and 1,430.2 per 10,000 females. Between 2000 and 2014, the values of the SEYLL indices decreased. In 2014 the SEYLL index was 3,750,249 for males and 2,379,030 for females, whereas SEYLL_p_ was 2,193.2 per 10,000 males and 1,269.4 per 10,000 females. In the fifteen-year study period, the average annual percentage change (AAPC) was −0.8% among males (95% CI −1.4 to −0.3) and −0.6% among females (95% CI −0.8 to −0.4).

**Table 1:** Standard expected years of life lost by sex and three broad cause groups, Poland, 2000–2014

	SEYLL		%		SEYLL_p_ (per 10,000)		AAPC	95% CI	
	2000	2014	2000	2014	2000	2014			
**Men**									
Group 1	212,025	188,778	4.9	5.0	114.4	101.4	−0.6	−2.2	1.1
Group 2	3,305,056	2,971,736	76.9	79.3	1,782.9	1,596.0	−0.8*	−1.5	−0.1
Group 3	780,654	589,735	18.2	15.7	421.1	316.7	−1.2*	−2.2	−0.2
Total	4,297,735	3,750,249	100.0	100.0	2,503.4	2,193.2	−0.8*	−1.4	−0.3
**Women**									
Group 1	145,087	122,635	5.4	5.1	73.6	61.8	−0.2	−0.9	0.5
Group 2	2,333,767	2,131,135	87.5	89.6	1,183.7	1,073.1	−0.7	−1.4	0.1
Group 3	189,263	125,260	7.1	5.3	96.0	63.1	−2.2*	−3.5	−0.9
Total	2,668,117	2,379,030	100.0	100.0	1,430.2	1,269.4	−0.6*	−0.8	−0.4

SEYLL–Standard Expected Years of life Lost. SEYLL_p_ - Standard Expected Years of life Lost per living persons. AAPC - Average Annual Percentage Change. CI–Confidence Interval. Group 1: Communicable, maternal, perinatal and nutritional conditions. Group 2: Non-communicable diseases. Group 3: Injuries. *p < 0.05.

**Figure 1:**
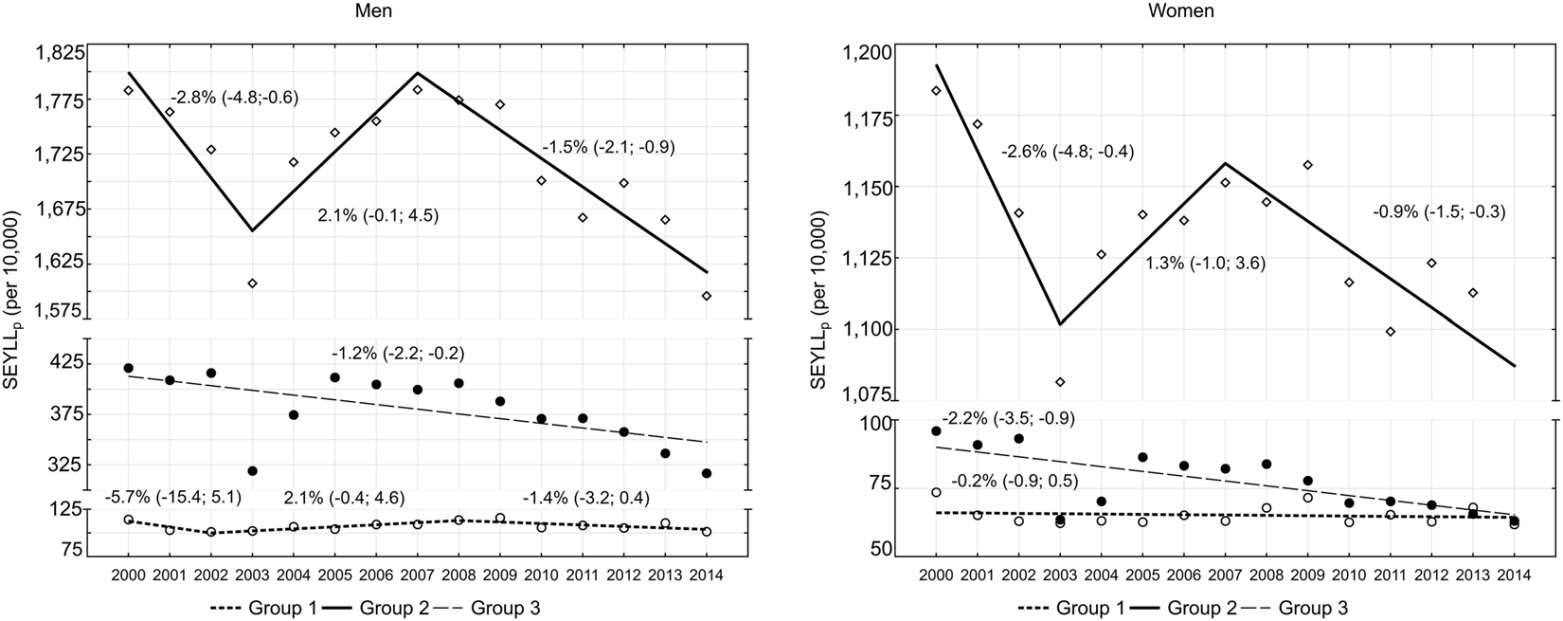
Time trends in SEYLL_p_ by three broad cause groups, Poland, 2000–2014.

Diseases from Group II contributed to the highest number of deaths. In 2000 chronic non-communicable diseases contributed to 76.9% of years of life lost among males (1,782.9 per 10,000) and 87.5% years of life lost among females (1,183.7 per 10,000). After a swift drop of SEYLL_p_ indices in the period 2000–2003, equal to −2.8% among males (95% CI −4.9 to −0.6) and −2.6% among females (95% CI −4.8 to −0.4), the indices quickly increased in 2003–2007 at the pace of 2.1% among males (95% CI −0.1 to 4.5) and 1.3% among females (95% CI −1.0 to 3.6). Between 2007 and 2014, the SEYLL_p_ values started to decline again and the average annual percentage change was −1.5% (95% CI −2.1 to −0.9) among males and −0.9% (95% CI −1.5 to −0.3) among females (Fig. 1). The AAPC for the chronic non-communicable diseases group was −0.8% among males (95% CI −1.5 to −0.1) and −0.7% among females (95% CI −1.4 to 0.1) in the fifteen-year study period.

In 2000, mortality caused by injuries (Group III) contributed to 18.2% of all years of life lost in males and 7.1% years of life lost in females. The SEYLL_p_ index was respectively 421.1 years per 10,000 males and 96.0 years per 10,000 females. Since 2000 the SEYLL_p_ index was gradually decreasing: APC = −1.2% among males (95% CI −2.2 to −0.2) and −2.2% among females (95% CI −3.5 to −0.9). In 2014 the values were: 316.7 years per 10,000 males and 63.1 years per 10,000 females.

Diseases included in Group I, i.e. communicable, maternal, perinatal and nutritional conditions, contributed to the lowest number of years of life lost. In 2000, the percentage was 4.9 in males and 5.4 in females, and in 2014 the values were 5.0 and 5.1 respectively. The SEYLL_p_ index for the year 2000 was 114.4 per 10,000 males and 73.6 per 10,000 females. Among males, this trend changed twice: in 2002 and 2008 (Fig. 1). Within the analysed 15 years, the AAPC was −0.6% among males (95% CI −2.2 to 1.1) and −0.2% among females (95% CI −0.9 to 0.5). In 2014 the values were 101.4 per 10,000 males and 61.8 per 10,000 females.

**Figure 2:**
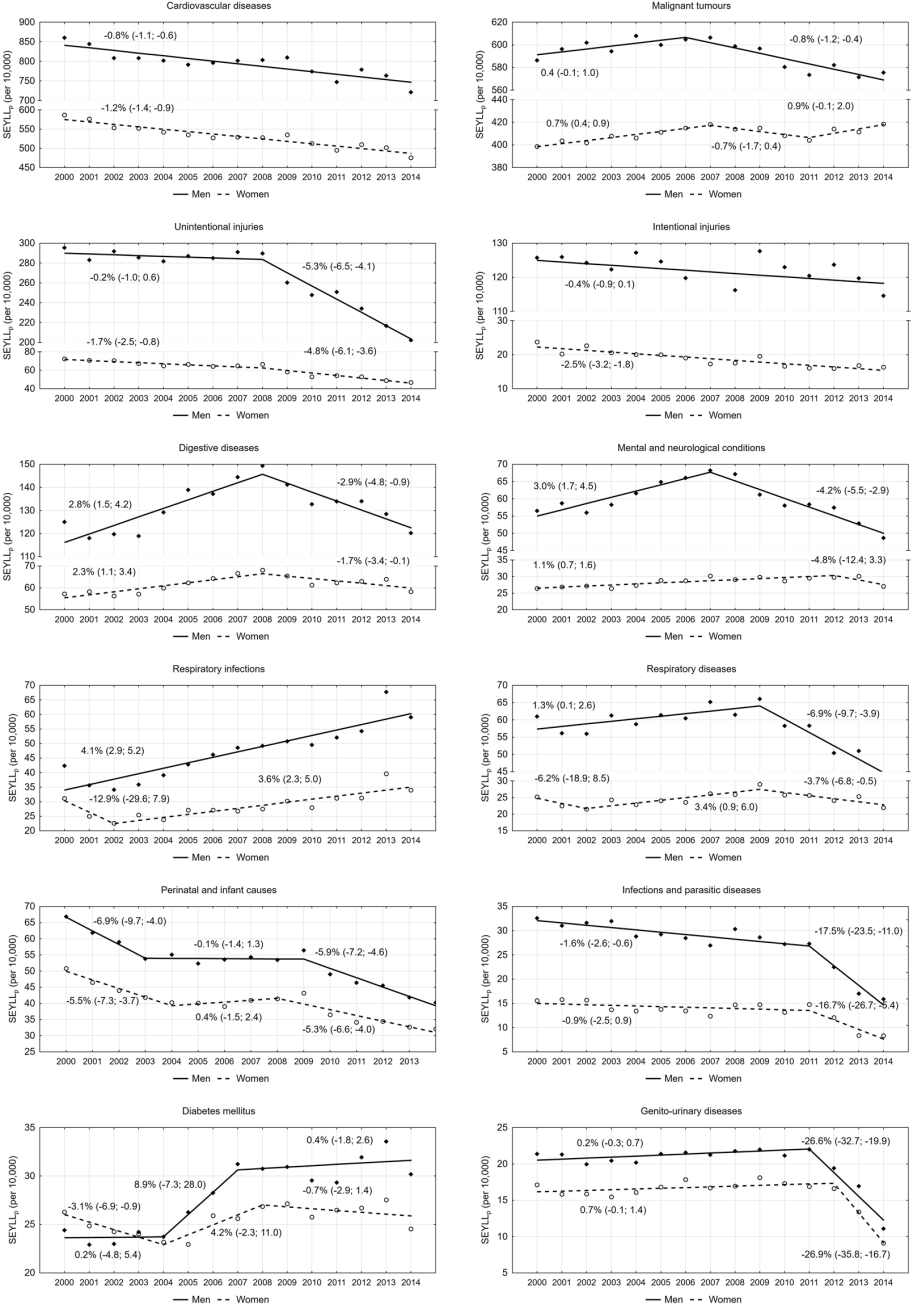
Time trends in SEYLL_p_ by main groups, Poland, 2000–2014.

A more detailed analysis of the main groups reveals that cardiovascular diseases and malignant tumours contribute to the greatest number of years of life lost (Table 2). It was noted that the AAPC for cardiovascular diseases was −0.8% among males (95% CI −1.1 to −0.5) and −1.2% among females (95% CI −1.4 to −0.9). The SEYLL_p_ values for malignant tumours were increasing between 2000–2007; from 2007 they started to decline. The decrease among males remained stable until 2014 (APC = −0.8%; 95% CI −1.2 to −0.4), but among females, the SEYLL_p_ started to grow in 2011 at the annual pace of 0.9% (95% CI −0.1 to 2.0) (Fig. 2).

**Table 2:** Standard expected years of life lost by sex and main groups, Poland, 2000–2014.

Cause of death	Men					Women				
	SEYLL_p_ (per 10,000)		AAPC	95% CI		SEYLL_p_ (per 10,000)		AAPC	95% CI	
	2000	2014				2000	2014			
Cardiovascular diseases	860.3	721.4	−0.8*	−1.1	−0.6	586.9	475.6	−1.2*	−1.4	−0.9
Malignant tumours	586.3	575.5	−0.3	−0.5	0.0	398.6	418.3	0.3*	0.0	0.7
Unintentional injuries	295.4	202.2	−2.4*	−3.0	−1.8	72.3	46.8	−3.0*	−3.7	−2.4
Intentional injuries	125.7	114.6	−0.4	−0.9	0.1	23.7	16.3	−2.5*	−3.2	−1.8
Digestive diseases	125.0	120.2	0.4	−0.6	1.4	57.3	58.3	0.5	−0.3	1.4
Mental and neurological conditions	56.5	48.7	−0.6	−1.5	0.2	26.4	27.0	0.3	−0.8	1.4
Respiratory infections	42.3	59.1	4.1*	2.9	5.2	31.1	34.0	1.1	−1.7	4.0
Respiratory diseases	61.0	43.5	−1.7*	−2.9	−0.5	25.2	22.0	−0.6	−2.8	1.6
Perinatal and infant causes	66.9	40.3	−3.7*	−4.5	−2.9	50.8	32.1	−3.4*	−4.2	−2.5
Infectious and parasitic diseases	32.6	15.9	−5.2*	−6.7	−3.7	15.6	8.3	−4.5*	−7.0	−1.9
Diabetes mellitus	24.4	30.2	2.1	−1.0	5.3	26.3	24.6	−0.1	−2.0	1.9
Genito−urinary diseases	21.4	11.1	−4.1*	−5.2	−3.0	17.1	9.1	−3.8*	−5.5	−2.1

SEYLL_p_ - Standard Expected Years of life Lost per living persons. AAPC - Average Annual Percentage Change. CI–Confidence Interval. *p < 0.05.

A substantial decrease in SEYLL_p_ values was observed for unintentional injuries–the third main factor contributing to a number of years of life lost. Since 2008, the APC was −5.3% among males (95% CI −6.5 to −4.1) and −4.8% among females (95% CI −6.1 to −3.6). The SEYLL_p_ index slightly decreased due to intentional diseases; the AAPC for males was only −0.4% (95% CI −0.9 to 0.1).

Regarding the number of years of life lost, positive trends were observed for digestive diseases, respiratory diseases, perinatal and infant causes, mental and neurological conditions as well as infections and parasitic diseases. After many years of a slight but gradual increase in the SEYLL_p_ index, its value suddenly declined for genito-urinary diseases. However, we should not draw hasty conclusions as the period of the decrease is short.

Regarding the number of years of life lost, the most negative trends were observed for respiratory infections. Between 2000–2014 the SEYLL_p_ index was gradually increasing in males and the annual decrease was 4.1% (95% CI 2.9 to 5.2). Among females, the index started to grow in 2002 and the annual increase was 3.6% (95% CI 2.3 to 5.0). Higher SEYLL_p_ index values among males were also observed for diabetes mellitus (AAPC = 2.1%, 95% CI −1.0 to 5.3).

## Discussion

The percentage of years of life lost in Poland and other European countries, calculated for three broad cause groups, is comparable^19–22^. Diseases included in Group II, i.e. chronic non-communicable diseases, including mainly cardiovascular diseases and malignant tumours, definitely contribute to the highest number of years of life lost. The pace and direction of changes in trends of SEYLL_p_ indices due to causes belonging to Group II changed twice in the period under study (Fig. 1), because of considerable differences concerning changes in years of life lost due to cardiovascular diseases and malignant tumours (Fig. 2). A separate analysis of trends for these two most important groups of death causes belonging to Group II indicates that the highest SEYLL_p_ values due to cardiovascular diseases were systematically decreasing within the fifteen-year study period. Positive changes in this respect accompanied constitutional transformations which occurred in Poland in the early 1990s. Social and economic changes contributed to changes in the lifestyle of Poles and improvement of the health care system^10^. The authors of an IMPACT model assessed that a decrease in mortality due to cardiovascular diseases resulted from the implementation of advanced treatment methods only in 37%, whereas in 50% it was due to favourable changes in cardiovascular diseases risk factors. The positive trends concerned a decrease in the total cholesterol level, which was related to changes in diet, a lowered systolic blood pressure among women and a decrease in smoking among males^23^. Poles became physically more active. However, current epidemiological studies show that that the positive trend has recently slowed down^24^. Alarmingly negative trends include a high incidence of obesity, diabetes and hypertension among males and an increase in smoking nicotine among females^23^. The impact of lifestyle-related risk factors on the number of years of life lost due to cardiovascular diseases was confirmed in a research study carried out in the Lodz region^10^. While there is a steady decreasing trend in Poland, the number of years of life lost due to cardiovascular diseases started to grow in the Lodz region since 2003. In 2013, SEYLL_p_ values in the Lodz region amounted to 847 per 10,000 males and 577 per 10,000 females and belonged to the highest in Poland^25^. On the basis of results obtained in a multi-centre nation-wide research study of the health situation of the population (WOBASZ) conducted in the period 2003–2005, it was found that only 5.4% of adults in the Lodz region had a healthy lifestyle, i.e. did not smoke tobacco, had a correct body weight, consumed fruit and vegetables every day and engaged in regular physical exercises. Among 3.8% of the study subjects not a single beneficial health behaviour out of the four mentioned above was observed^26^.

Smoking tobacco undoubtedly contributes to an increase in the number of years of life lost due to malignant tumours among females^11^. At the beginning of the 21^st^ century, about 43% males and 23% females in Poland smoked nicotine^27^. In 2014 the percentage of male smokers declined and it was 33%, whereas the percentage of female smokers remained approximately the same, i.e. 23%. Another serious problem is a growing number of young smokers. In 2010–2011, 23.9% of population aged 15–19 years were addicted to nicotine but in 2013–2014, the number of nicotine smokers in this age group grew and it was 38%^28^. Malignant tumours are the main cause of premature mortality (under 65 years of age). This phenomenon is particularly visible in the female population, where deaths due to malignant tumours make up 34% of the total number of deaths among all young females and above 48% among middle-aged women^29^. This fact directly results in a growing number of years of life lost among females due to this group of causes.

Group III, which is another broad cause group, includes unintentional injuries and intentional injuries. A number of years of life lost due to this cause was gradually decreasing among both males and females, because of a decrease in the SEYLL index values due to unintentional injuries, mainly including road traffic accidents. Standardized death rates (SDR) due to road traffic accidents in Poland are among the highest in Europe. In 2013 higher SDR values were observed only in Romania, Lithuania and Turkey^7^. The high level of mortality due to traffic accidents in Poland, as in other Eastern European countries, is caused by the poor quality of road infrastructure, poor technical condition of vehicles and dangerous behaviour of road users, mostly among men, who, above all, exceed speed limits and drive under the influence of alcohol^30^.

A far more negative trend concerns intentional injuries in males, where the number of years of life lost remained quite constant over the whole fifteen-year period. Results of studies indicate that the number of years of life lost is becoming increasingly diverse in different socioeconomic groups. Suicide is more and more often observed among less educated men and inhabitants of small towns and villages^12, 13, 31^. Since 2006, deaths due to suicide have outnumbered deaths due to traffic accidents. Similar tendencies are observed in other Eastern European countries, where socio-economic changes and an increased unemployment rate have led to the feeling of being deprived of their opportunities among many males, resulting often in social exclusion^32^. An increasing number of suicidal attempts is also related to alcohol abuse^33^. According to data of the Central Statistical Office in Poland, average consumption of pure alcohol per person has been gradually growing since 2000. In 2000, it was 7.1 litre, in 2014–9.4 litre. The greatest alcohol consumption is observed among males, aged 30–49 years, holding elementary or vocational education, inhabitants of small towns or villages^34^.

Mortality due to causes from Group I, which includes communicable diseases, maternal, perinatal and nutritional disorders, contributes to a relatively small number of years of life lost (about 5.0% of the total SEYLL value), both in Poland and in other highly developed European countries. However, trends in this group are not positive–since 2003 the APC among males has been increasing (APC = 2.1%), whereas among females–it has been decreasing. The decline, however, is statistically insignificant (APC = −0.2%). A more detailed analysis of causes indicates that deaths mainly due to respiratory infections, which include influenza and pneumonia, contribute to the above trends. Researchers dealing with this problem point out an alarmingly low (and decreasing) percentage of population vaccinated against influenza, which does not help to reduce morbidity. While the European Council recommends at least 75% of the population obtain the vaccination, in Poland for many years it has not exceeded even 5%. Moreover, despite the regular advancement in the influenza vaccination, the use of this vaccine has been gradually decreasing (vaccination on the level 3.4% in 2015 vs. 6.8% in 2009)^35^. On the other hand, the authors observed a distinct decrease in the SEYLL_p_ value due to infections and parasitic diseases after 2011. However, the decline appears to be a consequence of a rapid reduction of the number of deaths which were caused, as WHO wrongly classified, by septicaemia (A40 and A41 codes according to ICD–10). The disease which had preceded septicaemia should have been given as a real cause of death. Hence, it can be concluded that mortality due to infections and parasitic diseases has not really decreased but statistics regarding these deaths have changed^36^.

Statistical data with regards to years of life lost due to perinatal and infant causes have been systematically improving. Despite those positive trends, in 2014 the SDR due to infant mortality was higher than the mean value for the EU-28 (4.2 vs. 3.7 per 1,000 live births) and three times as high as in Cyprus, a country with the lowest infant mortality in Europe^7^.

## Methods

The study material includes a database which contains information gathered from 5,601,568 death certificates of inhabitants of Poland, who died between 2000 and 2014, provided by the Department of Information of the Polish Central Statistical Office. The given number refers to all deaths of Polish inhabitants which occurred in the 15-year study period.

Years of life lost were counted and analysed by the method described by *Christopher Murray* and *Alan Lopez* in *Global Burden of Disease* (GBD) study^37^. The SEYLL index (*Standard Expected Years of Life Lost*) is calculated from the expected remaining years, as specified by a normative survivorship that is derived from model life tables for the referential (standard) population. According to guidelines set forth by WHO experts, in the Global Burden of Disease (GBD) 2010 study, the authors applied tables of the average expected life span, based on the lowest mortality rate occurring in each age group, in countries with population above 5,000,000^38^.

For the purpose of the study the SEYLL index was calculated according to the following formula:

$${\boldsymbol{SEYLL}}=\sum _{{\boldsymbol{x}}=0}^{{\boldsymbol{l}}}{{\boldsymbol{d}}}_{{\boldsymbol{x}}}{{\boldsymbol{e}}}_{{\boldsymbol{x}}}^{\ast }$$

where ${{\rm{e}}}_{{\rm{x}}}^{\ast }$–standard expected years of life that remain to be lived in each age, based on GBD 2010 tables of the average expected life span, d_x_–a number of deaths in age x, x–age when the death occurred, l–the last year of age till the population lives.

The authors also calculated the SEYLL_p_ index (*per living person)*, where the absolute SEYLL value corresponded to the size of the Polish population in particular years^39^.

Death causes are coded according to the *International Statistical Classification of Diseases and Health Related Problems*–*Tenth Revision*–*ICD-10*. The original Global Burden of Disease study classified disease and injury causes using a hierarchical structure^40^. The first level of disaggregation comprised three broad cause groups: communicable diseases and maternal, perinatal and nutritional disorders (group I), chronic non-communicable diseases (group II) and all injuries (group III). The authors made a detailed analysis for 12 main groups of mortality: cardiovascular diseases (I00 –I99), malignant tumours (C00–C97), unintentional injuries (V01 – × 59, Y40–Y86, Y88, Y89), intentional injuries (X60–Y09, Y35–Y36), digestive diseases (K20–K92), mental and neurological conditions (F04–F99), respiratory infections (J00–J22, P23, H65–H66), respiratory diseases (J30–J98), perinatal and infant causes (P00–P96, except P23, P35, P37), infections and parasitic diseases (A00–B99, N70–N73, N30, N34, P35–P39, G00, G03, G04), diabetes mellitus (E10–E14) and genito-urinary diseases (N00–N29, N31–N33, N35–N64, N75–N98).

The analysis of time trends was carried out with *joinpoint* models and *Joinpoint Regression Program* (version 4.0.3 April 2013; Statistical Research and Applications Branch, National Cancer Institute)^41^. This method is an advanced version of linear regression, where time trend is expressed with a broken line, which is a sequence of segments joined in joinpoints. In these points, the change of the value is statistically significant (p < 0.05). The Grid Search method was chosen. Permutation tests were used to select the number of joinpoints. Fit of an uncorrelated errors model was used as autocorrelated errors option. The maximum number of joinpoints was 2. Minimum number of data points between two consecutive joinpoints was 4. We also calculated *annual percentage change* (APC) for each segment of broken lines and *average annual percentage change* (AAPC) for a full range of analysed years with corresponding 95% *confidence intervals* (CI).

## Limitations

The quality of analyses conducted with the use of mortality statistical data depends on complete and reliable information included in death certificates, but mainly on proper and precise presentation of death causes. The procedure of coding death causes in Poland is performed in a similar way to the majority of countries in the world, being based on the so-called underlying cause of death, or the disease which triggered a pathological process, leading to death. Poland is a country with 100% completeness of death registration, but the quality of cause of death coding was unsatisfactory, particularly in the case of cardiovascular diseases.

The World Health Organization tried to solve the problem of wrong coding of death causes by creating a list of so-called ‘garbage codes’, which should never be indicated as the primary death cause. The two ‘garbage codes’ most frequently used by doctors in the category of cardiovascular diseases are ‘heart failure’ (I50) and ‘generalized and unspecified atherosclerosis’ (I70.9)^42^.

Taking that in consideration, certain changes were introduced in Poland in 2009. In order to standardize death causes, which are subject to further statistical analyses, it was determined that the doctor who states the death is responsible for filling in the death card, into which he or she puts the primary, secondary and direct death cause, whereas qualified teams of doctors are responsible for coding death causes according to the ICD-10 classification. The duties of a dozen of regional statistical offices were taken over by the Polish Central Statistical Office. The relatively short time that the new system of processing data on deaths has been operating impedes its evaluation.
